# Agent-Based Modeling and Simulation (ABMS)on the influence of adjusting medical service fees on patients' choice of medical treatment

**DOI:** 10.1186/s12913-023-09933-3

**Published:** 2023-08-30

**Authors:** Danhui Li, Jia Yang, Jin Li, Ning Zhao, Wensheng Ju, Moning Guo

**Affiliations:** 1 Office of Medical Affairs, Shaanxi Provincial People’s Hospital, No. 256 Youyi West Road, Beilin District, Xi’an, 710068 Shaanxi China; 2 School of Public Health, Capital Medical University, No. 10 Xitoutiao, You’anmen Wai, Fengtai District, Beijing, 100069 China; 3 Beijing Municipal Health Big Data and Policy Research Center, No.277 Zhaodengyu Road, Beijing, 100034 China

**Keywords:** Differentiated medical service prices, Simulation model, Behavior of seeking medical services, Simulation experiment

## Abstract

**Background:**

We explored the impact of medical service fee adjustments on the choice of medical treatment for hypertensive patients in Beijing. We hope to provide decision-making reference to promote the realization of hierarchical diagnosis and treatment in Beijing.

**Methods:**

According to the framework of modeling simulation research and based on the data of residents and medical institutions in Beijing, we designed three models of residents model, disease model and hospital model respectively. We then constructed a state map of patients’ selection of medical treatment and adjusted the medical service fee to observe outpatient selection behaviors of hypertensive patients at different levels of hospitals and to find the optimal decision-making plan.

**Results:**

The simulation results show that the adjustment of medical service fees can affect the proportion of patients seeking medical treatment in primary and tertiary hospitals to a certain extent, but has little effect on the proportion of patients receiving medical treatment in secondary hospitals.

**Conclusions:**

Beijing can make adjustments of the current medical service fees by reducing fees in primary hospitals and slightly increasing fees in tertiary hospitals, and in this way could increase the number of patients with hypertension in the primary hospitals.

## Background

In August 2016, the National Health Conference of China put the hierarchical diagnosis and treatment system as the top of the five basic medical and health systems. The state has proposed to improve the medical and health service system, improve the capacity of grassroots medical and health services, guide public hospitals to participate in hierarchical medical treatment, and guide people's medical needs in a scientific and reasonable way. In order to promote the implementation of hierarchical diagnosis and treatment, the state launched a pilot program of graded diagnosis and treatment in 70% prefectures and cities in 2016, and expanded the pilot program to 85% prefectures and cities in 2017, taking chronic diseases as the starting point with the aim of achieving breakthrough in promoting hierarchical diagnosis and treatment. A difficult problem in the realization of hierarchical diagnosis and treatment policies is how to effectively guide patients [1]. The policy exerts major influence on the options of patients to seek medical treatment in hospitals at all levels. The adjustment of medical service prices is an effective way to guide the flow of chronic disease patients to the grass-roots level under the existing service capacity of primary medical institutions.

Due to the particularity of medical service, residents' demand for medical service and medical treatment behavior are different from ordinary goods [2]. Patients' choice of medical services is influenced by many factors, among which the most important factors include three types: propensity characteristics (demography, social structure, health beliefs), enabling resources (health policy, resources, organization), and needs (external environment, health status) [3]. Among them, the severity of the disease is the primary factor; the level of medical technology service is an important factor; the patient's demographic characteristics are secondary factors; and the medical insurance and medical service prices are the guarantee factors for achieving graded diagnosis and treatment [4]. Individual residents' medical treatment selection is a very complex process.

Many scholars have conducted in-depth studies on patients' medical treatment selection behavior. Kim-Sarah Krinke et al. based on literature search and qualitative research, used discrete choice experiment (DCE) to analyze and study primary health care preferences of eight rural populations in Germany. The results showed that patients' choice of medical treatment was influenced by factors such as whether to provide home visits, distance from clinics, clinic opening hours and clinic facilities, among which whether to provide home visits and clinic opening hours particularly affected rural patients' choice behaviors [5]. Chiara Seghieri et al. used mixed logistic regression to study the choice of non-emergency cardiology clinics for patients in Tuscany, Italy. The research focused on two factors: travel time and waiting time. The results show that the difference in patient choice depends on age and socioeconomic status, and patients prefer to go to the nearest hospital, because the waiting time is shorter [6]. Mohammad et al. surveyed 381 patients from 14 military hospitals in Tehran Province, Iran, and interviewed 22 managers and people in charge of outpatient wards. They analyzed the factors for patients to choose military hospitals from the perspective of patients and managers. The results show that "hospital diagnosis and treatment environment" and "doctors and employees" are among the most important factors to attract patients to seek medical treatment [7]. Yeon-Yong Kim et al. conducted an exploratory analysis of nine influencing factors that affected patients' medical treatment behavior based on a survey of 999 19–59-year-old Korean adults. The results indicated that patients preferred to choose a clinic based on the consideration of accessibility, the friendliness of medical staff, whether it is patient-centered, and they preferred to choose primary hospital based on the consideration of whether it is clean and hygienic, and they preferred to choose tertiary hospitals based on the consideration of the reputation and structural factors of the hospital [8]. From the studies of scholars in different countries on patients' medical treatment choice behaviors, it can be seen that patients' medical treatment choice behavior is affected by many factors. The structure and distribution of medical resources in different countries are different, and relevant medical policies have their own strengths. However, they all aim to guide patients to seek medical treatment rationally and promote the optimal allocation of medical resources.

In April 2017, Beijing fully implemented “the implementation plan for the separated management between medicine care and drug” [9], the core of which is to promote hierarchical diagnosis and treatment by abolishing drug markups and establishing medical service fee. Medical service fee referred to the outpatient treatment fee determined by the doctor's professional title level. There is a considerable gap in the price standard of medical service fees between different levels of medical institutions and different professional titles of doctor within a single medical institution. The establishment of medical service fee can compensate part of the operating cost of medical institutions and reflect the technical labor value of medical personnel. Moreover, the gap between the prices of different levels of medical services can guide the rational flow of patients, reshaping the distribution of patients among hospitals at all levels [10], so as to promote the realization of hierarchical medical treatment.

The hierarchical diagnosis and treatment policy in Beijing took initial effects. Compared with the same period in 2017, the number of outpatient emergencies in tertiary and secondary hospitals decreased by 11.9% and 0.4% respectively, the number of outpatient emergencies in primary hospitals and primary medical and health institutions in rural areas increased by 16.4%, and the number of diagnosis and treatment in some community health service institutions in urban areas increased by more than 20% [11].

In order to further promote the effect of the hierarchical diagnosis and treatment, this study explores the influence of the adjustment of medical service fees in different levels of hospitals on patients' medical choice behavior through the agent simulation model, so as to provide a reference for the government to promote the reform of hierarchical medical policy.

Based on literature studies and the actual situation of patients' options of medical treatment, this simulation study proposed the following assumptions:

- Assumption 1: Patients aim to get diseases cured to maximize their own utility.
- Assumption 2: The patient's disease is hypertension and is divided into three stages according to the severity of the disease.
- Assumption 3: All hospitals are public hospitals and designated hospitals for Medicare reimbursement.
- Assumption 4: Hospitals are divided into three types: primary, secondary and tertiary hospitals.
- Assumption 5: Except for different geographical locations, hospitals at the same level have no other differences.
- Assumption 6: Medical insurance reimbursement is not considered during experimental research.

## Methods

Faced with the complexity of health policy operation system, traditional qualitative and quantitative health policy research methods have certain limitations. As an experimental, procedural and prospective study, simulation method has brought a new perspective to the study of health policy and becomes one of the hot spots in the field of health research in recent years. Syed Salleh, Praveen Thokala and others [12] carried out a systematic review of the literature on the use of simulation modeling in healthcare research, and found that simulation modeling technology has been applied to medical decision-making widely. Simulation models can be used to help understand, explain, and predict the behavior of systems. However, traditional modeling methods focus on individuals rather than their interactions, so they are not applicable for understanding and interpreting the behavior of complex systems [13]. On the contrary, complex system methods, such as system dynamics, social network analysis, and modeling based on subject, explicitly model interactions and theoretically explain its existence and impact in the real world [13, 14]. Complex systems have several attribute definitions, including emergence, feedback, and adaptation. Emergence describes the characteristics of a complex system. It cannot be predicted directly from the elements inside the system, and it is not just a simple sum of the parts; feedback describes the situation where changes will strengthen or balance further changes; adaptation means behavioral adjustments based on interventions [15].

Agent-Based Modeling and Simulation (ABMS) is a methodological science for studying complex systems, and is associated with many other fields [16]. ABMS is centered on "agents" [17]. It is formulated around the creation of a set of objects (called "agents") that are placed in an environment and have personalized attributes and rules that interact with the environment and other agents [18]. This method has been applied to the fields of transportation, communication, marketing, medical and health care, and has become a new method to solve the problems of consumer behavior and policy analysis in the above fields [19].

Most modeling needs to be realized through programming software. Commonly used main body modeling software includes AnyLogic, Repast, Swarm, NetL-ogo and so on [20].Among them, the software AnyLogic is developed by the Russian company XJ TechnologieS. Its modeling language is Java, with a high degree of visualization. It is an efficient and practical modeling software that can fully support Agent-Based Modeling and Simulation, discrete event modeling, and system dynamics modeling [21]. Some scholars have used this method to study the patients' medical choice behavior. Scholars Zhang Yu et al. used the research methods of system dynamics and Multi-Agent modeling to analyze the medical behavior of the rural population, which provided methodological reference and decision-making basis for solving the imbalance of rural medical resources [22]. Meng Fanyuan et al. used the Anylogic software platform to analyze the macro-impact of health insurance policy adjustments on Shenyang patients' medical choice behavior [23]. Tan Min et al. analyzed the impact of medical insurance policy adjustment on the choice of medical treatment and the flow direction of medical insurance funds for inpatients with hypertension in Beijing based on Agent-Based Modeling and Simulation [24]. Abdullah Alibrahim et al. studied the impact of patients' medical choices on medical costs, medical quality and providers through Agent-Based Modeling and Simulation [25]. These researchs provide methodological reference for this study.

In this study, Anylogic was used to build the agent-based model. We established corresponding features and rules for the model without modifying the model structure [16], and dynamically tracked and recorded the macro data of patient's choice of medical treatment under the running of model throughout the simulation time.

### Experimental research design

In this study, Anylogic 8.0 simulation platform was used to establish an agent-based patient medical selection model, in order to simulate option of patient's medical treatment under the specific graded diagnosis and treatment policy. By constructing the patient model, the disease model and the hospital model, determining the input and output variables of the model, and designing the patient's medical treatment selection process and corresponding assumptions and rules, we dynamically recorded and tracked the macro data displayed under the patient's medical treatment selection model operation through the running of the model, and explored the interaction between hierarchical diagnosis and treatment policies and patient behavior. We used the real data of Beijing hospitals and individual residents during the simulation, and scaled the data by 10000: 1.

#### Research object

Based on the country's current policy to promote hierarchical diagnosis and treatment of chronic diseases such as hypertension and diabetes, the hypertensive outpatient patients covered by medical insurance for urban residents in Beijing were selected in this study. The screening criteria are: ① being covered by Beijing urban residents basic medical insurance, ② outpatients in primary, secondary and tertiary hospitals in Beijing, ③ being over 35 years old.

#### Data source

The basic demographic data of Beijing residents were obtained from Beijing bureau of statistics. The data of the secondary and tertiary hospitals were from the statistical data of the Beijing Municipal Health Commission Information Center from 2015 to 2017. The data of the tertiary hospitals were from the first page of outpatient medical records, and the data of the secondary hospital were from 30 secondary hospitals managed by the information center, and the data of the primary hospitals (community health service center) were mainly based on the data of three district social management centers.

#### Model setup

In the ABMS, residents were abstracted as agents, and five rules were designed, including the limitation of patients' medical treatment cost, the limitation of medical institutions' geographical location, the limitation of medical service capacity, the preference of medical service institutions, and the policy factor of medical service fee adjustment. Under the constraints of models and rules, residents finally completed the process of medical treatment selection through constantly adjusting medical service fees.

### Run of simulation experiment

#### Resident model

The residents in the simulation experiment were initially set at 2022 number based on the resident population data in 2015–2017 in Beijing, and the numbers were scaled by 10,000: 1. The resident model mainly considered the patient's age, income, disease situation, distance. In this model, the following rules were designed for the Agent:

- Rule 1: Restrictions on medical expenses. After the patient was ill, he could only receive treatment if he had the ability to pay. Therefore, it was assumed that each Agent had an income every year and satisfies the lognormal distribution ($\mathrm{lgIncome}\sim \mathrm{N}(\upmu ,{\mathrm{\vartheta }}^{2})$) among the population. In reality, the income of residents for medical treatment was limited, so there was a proportional coefficient ($\mathrm{\alpha }$) of medical expenses. Only when:$\mathrm{Income \alpha }>{\mathrm{D}}_{\mathrm{cost}}$, the patient could choose medical treatment, and ${\mathrm{D}}_{\mathrm{cost}}$ was the average medical cost of hypertension.
- Rule 2: The geographical location of the hospital was restricted. Assuming that each patient had a geographical location restriction to the doctor: Distancemax. Only when Distance ≤ Distancemax, and $\mathrm{Income \alpha }>{\mathrm{D}}_{\mathrm{cost}}$, the patient could receive treatment. Considering the severity of the condition and the fact that most patient would like to choose higher lever hospital, the hospital search range of each patient could be increased; that is, the expansion coefficient K (K ≥ 1) was introduced to Distancemax, so that Distance ≤ K*Distancemax (first stage of disease K = 1, second stage disease K = 2, third stage disease K = 3). Distancemax = 30 km.

#### Hospital model

The number of hospitals were set at a ratio of 1:200 to the actual number, and the number of beds were set at 1:50. Finally, 65 hospitals were included, including 40 primary hospitals, 17 secondary hospitals, and 8 tertiary hospitals.

The hospital model mainly took into consideration the location, ability to accommodate patients (capabililty), average medical cost of hypertension (Dcost), and average treatment time (Dtime) of the medical institution.

The medical service institutions in this study were tertiary hospitals (including general hospitals and specialty hospitals), secondary hospitals and primary hospitals. Generally speaking, the medical conditions of tertiary hospitals were better than those of secondary hospitals, and secondary hospitals were superior to primary hospitals. Moreover, the price of medical services of the same type of diseases was highest in tertiary hospitals, followed by secondary hospitals and primary hospitals. The number of tertiary hospitals was smaller than that of secondary hospitals, whose number was smaller than that of primary hospitals. In addition, each hospital would have its own geographic location, ability to accommodate patients, and time for curing a certain type of disease.

Therefore, for each medical service institution, a quintuple could be used to represent the hospital (xLocation, yLocation, Hcapability, Dcost, Dtime). xLocation and yLocation represented the vertical and horizontal coordinates of the hospital's geographic location, and Hcapability represented the medical institution's treatment of a certain type of disease capacity. In the classification of medical institutions, the number of hospital beds was one of the indicators to measure the capacity of the hospital. The number of patients that could be accommodated for a certain type of disease per unit time was chosen to represent Hcapability. Hcapability = 0 means the medical service agencies did not have the ability to treat such diseases. Dcost represented the average medical cost required for each treatment of this type of disease, and Dtime represented the average time spent in treating a certain type of disease.

In the hospital model, the following rules were designed for medical institutions:

- Rules: Medical service capacity restrictions. Hcapability was used to indicate the number of patients (ie the number of beds) that a certain type of disease could accommodate in each hospital at the same time. When the patient met rules 1 and 2 in the Resident model, the optional hospital Hcapability > 0.

#### Disease model

**Fig. 1:**
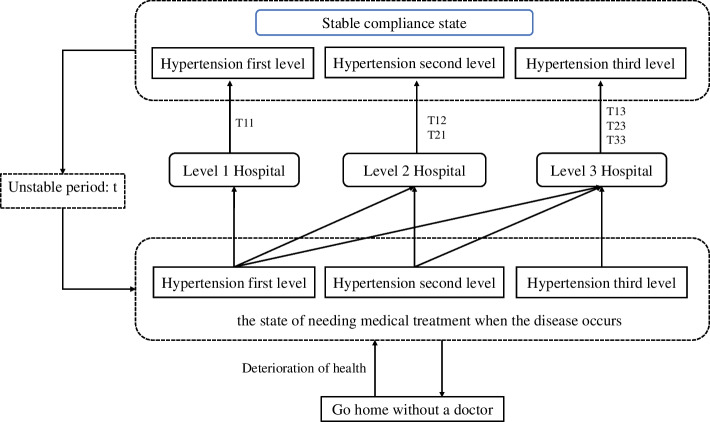
Disease model medical care selection diagram

Combined with the resident model, the entire population was divided into a diseased state and a healthy state. The prevalence of hypertension patients could be divided into first, second and third grades according to the severity of the disease. The hypertension patients had three states in the model: stable compliance state, unstable state and disease state. There were two destinations in the model: ①go to hospital for treatment, ②stay at home without any treatment. This study assumed that these three levels of hypertensive patients would be back at the original level after medical treatment; that is, the treatment of hypertensive patients did not deteriorate their condition. In addition, patients with first-degree hypertension could go to first-, second-, and tertiary hospitals; patients with second-degree hypertension could go to secondary and tertiary hospitals; patients with third-degree hypertension could only go to tertiary hospitals. Different levels of hospitals had different time frames of treatment for patients with different levels of hypertension: the treatment time frames required for patients with first-grade hypertension in primary, secondary, and tertiary hospitals are T11, T12, and T13. The treatment time frames required for patients with second-grade hypertension in secondary and tertiary hospitals for patients with high blood pressure are T22 and T23. The treatment time frames required for patients with third-degree hypertension in tertiary hospitals is T33. These three grades of hypertensive patients reached a stable compliance state after treatment, and then entered the disease state again after the unstable period of t days. See Fig. 1 for details.

#### Policy adjustable parameters

Most hypertensive patients would like to choose to go to general clinic for medical treatment because they mainly wanted to prescribe medicine. So this research would adjust the medical service fee for the general outpatient clinic. We took the standard of general outpatient medical service fee of hospitals at different levels in Beijing as the initial value. The initial value was 20 yuan for the primary hospitals, 30 yuan for the secondary hospitals, and 50 yuan for the tertiary hospitals. The adjustment range was 2 yuan, and the medical service fee was tertiary ≥ secondary ≥ primary.

#### Model test

In order to ensure the validity of the simulation data, the simulation model needed to be tested by comparing the simulation data of the main observed variables with the actual data to calculate the relative error of the variables (Table 1).

**Table 1:** Validation of the applicability of the simulation model (%)

Grade of hospital	Truthful data	Simulation data	Relative error	RMS
Primary hospitals	35.82	40.77	-13.81	10.53
Secondary hospitals	32.61	31.12	4.55	
Tertiary hospitals	31.57	28.11	10.97	

As can be seen from the table, the RMS value is 10.53%, close to 10%, close to acceptable range

## Results

In this study, a total of 7 experiments were designed, including adjusting the medical service fee of a certain level alone and adjusting the medical service fee of different levels of hospital simultaneously. The adjustment range of each medical service fee was 2 yuan, and 270 simulation experiments were conducted.

Medical service fees of primary hospital adjusted individually or simultaneously: The proportion of patients in primary hospitals decreased significantly, the proportion of patients in tertiary hospitals increased significantly, and the proportion of patients in secondary hospitals did not change significantly.

### Only adjusted the medical service fee of primary hospital

When only the medical service fee of the primary hospital was adjusted, and the fee adjustment ranged from 6 to 20 yuan, we found that although the fluctuation of the proportion of patients seeking medical treatment in primary and tertiary hospital was not big, the proportion for primary hospital patients slightly decreased, and ratio for tertiary hospital patients slightly increased. When the medical service fee of primary hospital was adjusted to 14 yuan, the proportion of patients in primary hospital was the largest, which as 40.99%. The proportion of patients in the tertiary hospitals was the lowest, which was 27.77%. When the medical service fee of the primary hospital was adjusted from 20 to 22 yuan, the proportion of patients in the primary hospital has decreased significantly, and the proportion of patients in the tertiary hospital has increased significantly, which became more than that of patients in secondary hospitals, and after that tertiary hospital was more flat. The proportion of visits to the secondary hospitals was basically unchanged when the medical service fees of the primary hospitals were adjusted. It showed that increasing the medical service fee in primary hospitals would reduce the proportion of patients seeking medical treatment in primary hospitals and increase the proportion of patients seeking medical treatment in tertiary hospitals. When the medical service fee of the primary hospital was adjusted individually to 14 yuan, the proportion of patients in the primary and tertiary hospitals reaches the optimal condition under the experimental conditions. The proportions of patients in different levels of hospitals after the medical service fees of primary hospital were adjusted could be seen in Table 2.

**Table 2:** The proportion of patients seeking medical treatment after the medical service fees of primary hospitals were adjusted

Proportion of patients in different levels of hospitals after the medical service fees of primary hospitals were adjusted (medical service fee: RMB; Percentage: %)													
Medical service fees	6	8	10	12	14	16	18	20	22	24	26	28	30
Primary hospitals	40.79	40.87	40.74	40.46	40.99	40.53	40.1	40.77	34.85	34.58	35.2	34.88	35.12
Secondary hospitals	31.29	31.32	31.32	31.16	31.23	31.59	31.47	31.12	31.37	31.71	30.99	31.59	31.08
Tertiary hospitals	27.91	27.81	27.94	28.38	27.77	27.88	28.43	28.11	33.78	33.71	33.81	33.53	33.80

### The medical service fees of primary and secondary hospitals adjusted simultaneously

**Fig. 2:**
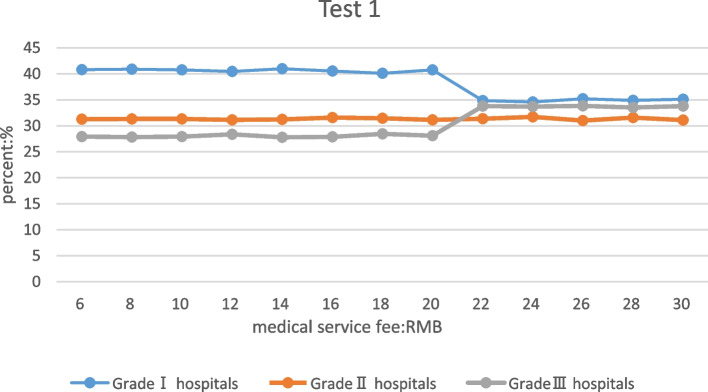
Patient's choice of medical treatment in hospitals of different levels after adjusting the medical service fee of primary hospitals

Table 3 shows the proportion change of patients in different levels of hospitals after the medical service fees of primary and secondary hospitals were adjusted. The changing trend of patients’ proportion when the service fees of primary and secondary hospitals were adjusted was the same as the trend when the service fee of primary hospitals was adjusted individually. But the change nodes of the medical service fee were different. When the medical service fee of primary and secondary hospitals were 12 yuan and 22 yuan, the proportion of patients in the primary hospital reached the highest, which was 40.81%. When the medical service fee of the primary and secondary hospitals were 16 yuan and 26 yuan, the proportion of patients in the tertiary hospitals was the lowest, which was 27.58%. To achieve hierarchical diagnosis and treatment, the medical service fee was better set at 16 yuan and 26 yuan in primary and secondary hospitals. Table 3 already shows the change in the proportion of visits under different medical service fees, so the graph for test 4 will not be shown (test4 the trend of medical flow in different hospitals is the same as Fig. 2).

**Table 3:** The proportion of patients seeking medical treatment after the medical service fees of primary and secondary hospitals were adjusted

The proportion of patients in different levels of hospitals after the medical service fees of primary and secondary hospitals were adjusted (medical service fee: RMB; Percentage: %)											
Medical service fees	10	12	14	16	18	20	22	24	26	28	30
	20	22	24	26	28	30	32	34	36	38	40
Primary hospitals	40.1	40.81	40.58	40.47	40.53	40.77	34.88	34.89	35.46	34.32	35.14
Secondary hospitals	31.42	33.55	31.40	31.95	31.56	31.12	31.33	31.13	31.41	31.41	31.22
Tertiary hospitals	28.47	30.16	28.03	27.58	27.91	28.11	33.79	33.97	33.12	34.28	33.63

### Adjusted simultaneously the medical service fees of primary and tertiary hospitals

Table 4 shows the proportion change of patients in different levels of hospitals after the medical service fees of primary and tertiary hospitals were adjusted. The changing trend of patients proportion when the service fees of primary and tertiary hospitals were adjusted was the same as the trend when the service fee of the primary hospitals was adjusted individually. But the change nodes of the medical service fee were different. When the medical service fee of the primary and tertiary hospitals were 16 yuan and 46 yuan, the proportion of patients in the primary hospital reached the highest, which was 41.24%, and the proportion of patients in the tertiary hospitals reached the lowest, which was 27.70%. It showed that under this setting of medical service fee level, the proportion of patients in the primary hospital has reached the optimum under experimental conditions. Table 4 already shows the change in the proportion of visits under different medical service fees, so the graph for test 5 will not be shown(test5 the trend of medical flow in different hospitals is the same as Fig. 2).

**Table 4:** The proportion of patients seeking medical treatment after the medical service fees of primary and tertiary hospitals were adjusted

The proportion of patients in different levels of hospitals after the medical service fees of primary and tertiary hospitals were adjusted (medical service fee: RMB; Percentage:%)											
Medical service fees	10	12	14	16	18	20	22	24	26	28	30
	40	42	44	46	48	50	52	54	56	58	60
Primary hospitals	39.63	40.48	40.44	41.24	40.27	40.77	40.88	34.69	35.22	35.21	34.69
Secondary hospitals	31.82	31.66	31.40	31.06	31.35	31.12	31.26	31.66	31.49	31.34	31.57
Tertiary hospitals	28.54	27.86	28.16	27.70	28.38	28.11	27.86	33.65	33.29	33.45	33.73

### Medical service fees of first- second-and tertiary hospitals adjusted simultaneously

Table 5 shows the proportion change of patients in different levels of hospitals after the medical service fees of primary, secondary and tertiary hospitals were adjusted. The changing trend of the patients proportion when the service fees of primary, secondary and tertiary hospitals were adjusted was consistent with the trend when the service fee of the primary hospitals was adjusted individually. But the medical service fee changing nodes are different. When the medical service fees of hospitals at all levels were 10 yuan, 20 yuan, and 40 yuan, the proportion of patients in the primary hospitals was the highest, which was 41.06%. When the medical service fee of each level hospital were 22 yuan, 32 yuan, and 52 yuan, the proportion of patients in the tertiary level hospital was the lowest, which was 27.61%. Calculate the difference of patient proportion under the two medical service charges respectively, 0.19%(41.06%-40.87%) for the primary hospitals and 0.11%(27.72%-27.61%) for the tertiary hospitals. For achieving graded diagnosis and treatment, when the medical service fees of primary, secondary and tertiary hospitals were set at 10 yuan, 20 yuan, and 40 yuan, was the best. Table 5 already shows the change in the proportion of visits under different medical service fees, so the graph for test 7 will not be shown (test7 the trend of medical flow in different hospitals is the same as Fig. 2).

**Table 5:** The proportion of patients seeking medical treatment after the medical service fees of primary, secondary and tertiary hospitals were adjusted

The proportion of patients in different levels of hospitals after the medical service fees of primary, secondary and tertiary hospitals were adjusted (medical service fee: RMB; Percentage:%)											
Medical service fees	10	12	14	16	18	20	22	24	26	28	30
	20	22	24	26	28	30	32	34	36	38	40
	40	42	44	46	48	50	52	54	56	58	60
Primary hospitals	41.06	40.51	40.09	40.09	40.46	40.77	40.87	35.16	34.80	35.46	34.87
Secondary hospitals	31.22	31.13	31.43	31.62	31.27	31.12	31.52	31.24	31.23	31.24	31.63
Tertiary hospitals	27.72	28.37	28.48	28.29	28.27	28.11	27.61	33.60	33.97	33.30	33.50

The medical service fee of the tertiary hospitals adjusted individually and those of the second and tertiary hospitals adjusted simultaneously: the proportion of patients in the primary hospitals increased significantly, the proportion of patients in the tertiary hospitals decreased significantly, and the proportion of patients in the secondary hospitals did not change significantly.

### Only adjusting medical service fees in tertiary hospitals

When the medical service fees of tertiary hospitals were adjusted to be between 30–48 yuan, the proportion of patients in primary, secondary and tertiary hospitals changed slowly, and the proportion of patients in primary hospitals was higher than that in tertiary hospitals. When the medical service fee was adjusted to rist from 48 to 50 yuan, the proportion of patients in the primary hospitals increased significantly, and the proportion of patients in the tertiary hospitals decreased significantly. When the medical service fee was adjusted to be between 50–60 yuan, the proportion of patients in the primary, secondary, and tertiary hospitals changed slowly, too, and the proportion of patients in the secondary hospital was higher than that in the tertiary hospital. The results show that the current setting of medical service fees in tertiary hospitals was reasonable. Throughout the adjustment range, the proportion of patients in secondary hospitals remained basically unchanged. See Table 6 for details.

**Table 6:** The proportion of patients seeking medical treatment after the medical service fees of tertiary hospitals were adjusted

The proportion of patients in different levels of hospitals after the medical service fee of tertiary hospitals was adjusted (medical service fee: RMB; Percentage: %)																
Medical service fees	30	32	34	36	38	40	42	44	46	48	50	52	54	56	58	60
Primary hospitals	35.18	34.94	34.88	34.67	34.99	34.59	35.47	35.04	35.09	34.61	40.77	41.45	40.43	40.26	41.04	40.38
Secondary hospitals	31.15	31.58	31.27	31.37	30.99	31.44	30.93	31.27	31.46	31.36	31.12	31.19	31.44	31.46	31.16	31.32
Tertiary hospitals	33.67	33.48	33.85	33.96	34.02	33.97	33.60	33.69	33.44	34.03	28.11	27.36	28.13	28.28	27.80	28.31

Table 6 shows the proportion change of patients in different levels of hospitals after the medical service fees of tertiary hospitals were adjusted. When the medical service fee of the tertiary hospital was 52 yuan, the proportion of patients in the primary hospital was the highest, which was 41.45%, and the proportion of patients in the tertiary hospital was the lowest, which was 27.36%. This shows that under this setting of medical service fee, the proportion of patients has reached the optimum under experimental settings.

### The medical service fees in secondary and tertiary hospitals adjusted simultaneously

**Fig. 3:**
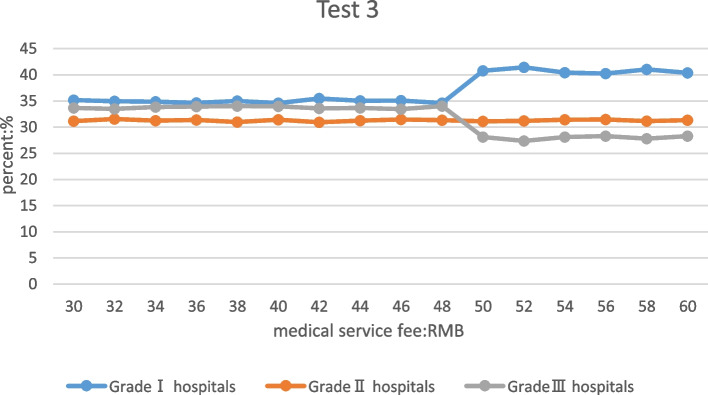
Patient's choice of medical treatment in hospitals of different levels after adjusting the medical service fee of tertiary hospitals

Table 7 shows the proportion change of patients in different levels of hospitals after the medical service fees of secondary and tertiary hospitals were adjusted. When the secondary and tertiary medical service fees were 28 yuan and 48 yuan, the proportion of patients in the primary hospital was the highest, which was 41.15%, and the proportion of patients in the tertiary hospital was the lowest, which was 27.57%. This shows that under this setting of medical service fee, the proportion of patients has reached the optimum under experimental conditions. Table 7 already shows the change in the proportion of visits under different medical service fees, so the graph for test 6 will not be shown (test6 the trend of medical flow in different hospitals is the same as Fig. 3).

**Table 7:** The proportion of patients seeking medical treatment after the medical service fees of secondary and tertiary hospitals were adjusted

The proportion of patients in different levels of hospitals after the medical service fees of secondary and tertiary hospitals were adjusted (medical service fee: RMB; Percentage: %)											
Medical service fees	20	22	24	26	28	30	32	34	36	38	40
	40	42	44	46	48	50	52	54	56	58	60
Primary hospitals	34.92	35.37	34.55	40.76	41.15	40.77	40.31	40.38	40.02	39.39	40.56
Secondary hospitals	31.41	31.18	31.32	31.63	31.28	31.12	31.57	31.01	31.66	31.82	31.41
Tertiary hospitals	33.67	33.45	34.13	27.61	27.57	28.11	28.13	28.61	28.31	28.79	28.03

**Fig. 4:**
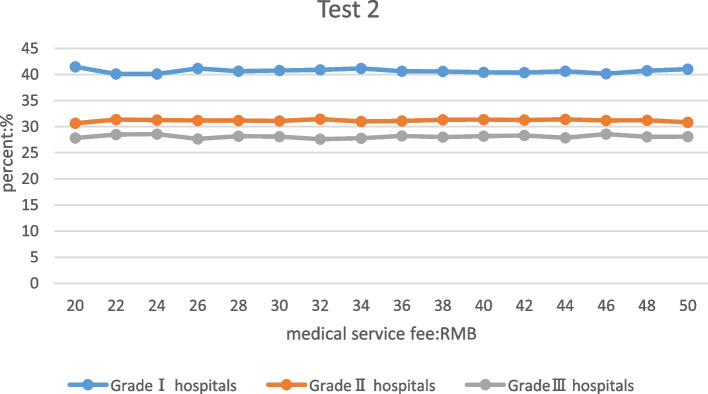
Patient's choice of medical treatment in hospitals of different levels after adjusting the medical service fee of secondary hospitals

The medical service fee of secondary hospitals adjusted individually: there was no significant change in the proportion of patients in different levels of medical institutions (Fig. 4).

Overall, the proportion of patients at all levels of hospitals changed relatively gently when the medical service fees of the secondary hospitals were adjusted. When the fees were adjusted from 20 to 22 yuan, the proportion of patients in the primary hospitals decreased slightly, and the proportion of patients in secondary and tertiary hospitals increased slightly. It shows that adjusting the medical service fees of the secondary hospitals had little effect on the proportion of patients at all levels of hospitals.

According to the experimental results, the flow direction of patients seeking medical treatment in different level hospitals appeared to show three trends: The first trend was the proportion of patients in the primary hospitals gradually decreased, the proportion of patients in the tertiary hospitals gradually increased, and the flow of patients in the secondary hospitals did not change, corresponding to the research results of tests 1, 4, 5, and 7. The second trend was the proportion of patients in the primary hospitals gradually increased, the proportion of patients in the tertiary hospitals gradually declined, and the flow direction of patients in the secondary hospitals did not change, corresponding to the research results of tests 3 and 6. The third trend was that there was no obvious change in the trend of medical treatment flow direction of patients at all levels of hospitals, corresponding to the results of test 2. The details are shown in Table 8.

**Table 8:** 7 groups of experimental optimal medical service fee classification summary

Research results	Test	Adjust the situation	The medical service fee for the best visit ratio for each grade of hospital
The proportion of patients seeking medical treatment in primary hospitals gradually decreased, the proportion in tertiary hospitals gradually increased, while the flow direction of patients seeking medical treatment in secondary hospitals remained unchanged	Test1	Adjustment of primary hospital medical service fees	Primary hospital: 14 yuan
	Test4	Adjustment of primary and secondary hospital medical service fees	Primary hospital: 16 yuan secondary hospital: 26 yuan
	Test5	Adjustment of primary and tertiary hospital medical service fees	Primary hospital: 16yuan Tertiary hospital: 46yuan
	Test7	Adjustment of primary, secondary and tertiary hospital medical service fees	Primary hospital: 10yuan Secondary hospital: 20yuan Tertiary hospital: 40yuan
The proportion of patients in primary hospitals gradually increased, the proportion in tertiary hospitals gradually decreased, while the flow direction of patients in secondary hospitals remained unchanged	Test3	Adjustment of tertiary hospital medical service fees	Tertiary hospital: 50 yuan
	Test6	Adjustment of secondary and tertiary hospital medical service fees	Secondary hospital: 26yuan Tertiary hospital: 46yuan
There was no significant change in the trend of the flow direction of patients to hospitals at all levels	Test2	Adjustment of secondary hospital medical service fees	Nothing

## Discussion

The results of simulation experiments show that the adjustment of medical service fees had an impact on the flow direction of patients for medical treatment, and there are certain rules. When we adjusted medical service fees of different levels of hospitals, there would be three types of changes in the flow direction of patients for medical treatment. The first law was: no matter when the medical service fee of the primary hospital was adjusted individually or simultaneously, the proportion of patients in the primary hospital would gradually decrease, the proportion of patients in the tertiary hospital would gradually increase, and the flow direction of patients to seek medical treatment in secondary hospitals would not change obviously. The second law was: without any adjustment of the medical service fee of the primary hospital, when we adjusted individually or simultaneously the medical service fee of secondary and tertiary hospitals, the proportion of patients in the primary hospital would gradually increase, the proportion of patients in the tertiary hospital would gradually decrease, and there was no obvious change in the flow direction of patients to seek medical treatment in secondary hospitals. The third law was: without any adjustment of the medical service fees of the primary and tertiary hospitals, when we adjusted individually the medical service fees of secondary hospitals, there would be no obvious change in the flow direction of patients to seek medical treatment at all levels of hospitals. In the first and second rules, with the change of medical service fees in hospitals of different levels, the flow of patients' medical treatment will also fluctuate to varying degrees. By looking for the nodes changes of the patient's medical treatment flow direction, we could find the best range of medical service charge adjustment and provide some policy suggestions for the promotion of graded diagnosis and treatment.

In response to the third law, in the premise that there is little difference in medical service fees between different levels of medical institutions and residents have the freedom to choose, residents have developed a preference for higher-level hospitals, and common diseases that can be solved in primary medical institutions are "siphoned" to tertiary hospitals [26]. In addition, a study has confirmed that tertiary hospitals "siphon" mainly target secondary hospitals rather than primary hospitals [27]. Tertiary hospitals have been in a favorable position in the competition, while primary hospitals have been supported by national policies, and secondary hospitals are in a " sandwich " predicament [28]. Secondary hospitals are squeezed by tertiary and primary hospitals in the competition, and are generally in an awkward position with an unfilled workload and unclear positioning, thus leading to no significant change in the flow of patients to secondary hospitals.

### Determine reasonable range of the medical service fees, which could attract patients to the primary hospitals

The simulation results show that the proportion of patients in primary hospitals would gradually decrease when the medical service fees of primary hospitals were increased individually or simultaneously. Therefore, how to determine reasonable range of medical service fee was the key of effectively attracting patients to the primary hospitals. After the proportion of patients in primary hospitals was analyzed, no matter how the service fees of secondary and tertiary hospitals were adjusted, the more reasonable range of the medical service fees in primary hospitals were 10 yuan-16 yuan, under which the proportion of patients in primary hospitals was at a high level. “The implementation plan for the separated management between medicine care and drug” began to be implemented in Beijing. The medical service fee of the general number in primary hospitals was determined to be 20 yuan in the implementation plan, which was slightly higher than the results of this study. Some Chinese scholars have concluded in their studies that the medical service fee policy would affect the patients' medical treatment flow direction. Yu Fei et al. [29] concluded that medical service fees had a positive guiding effect on medical behavior: Through economic leverage, a large number of patients would no longer blindly seek medical treatment in tertiary hospitals, and the idea of "small diseases should be treated by big experts" would be changed to promote tertiary hospitals to return to their functional position in the treatment of difficult critical diseases, which is conducive to the continuous promotion of hierarchical diagnosis and treatment.

### Adjust appropriately the medical service fees of secondary and tertiary hospitals, which could guide patients from secondary and tertiary hospitals to primary hospitals

In the simulation experiment, when the medical service fee of the tertiary hospitals was adjusted, or adjusted at the same time with the medical service fee of the secondary and tertiary hospitals, the proportion of patients in the primary hospitals would increase, while the proportion of patients in the tertiary hospitals would decrease gradually. This shows that the realization of hierarchical diagnosis and treatment could be promoted by adjusting the medical service fees of the secondary and tertiary hospitals, too. The research results of several groups of experiments showed the proportion of patients in primary hospitals was higher when the medical service fee of the tertiary hospital was no less than 50 yuan at the range between 50 yuan -54 yuan, and the medical service fee of the secondary hospital was not less than 26 yuan at the range between 26 yuan -30 yuan. Zhou Shuduo et al. analyzed the impact of the comprehensive reform of Beijing's “the implementation plan for the separated management between medicine care and drug” influence on the flow direction of outpatient and emergency patients. The results showed that the average number of emergency department visit per institution in the tertiary hospitals had a significant downward trend compared with the number at the same period last year after the reform, and the number of outpatient and emergency department visit per primary medical institution had an obvious upward trend compared with the number at the same period [30]. Their research indicated that the medical service fee policy prompted patients to be reasonably diverted to a certain extent.

### Coordinate multiple policies such as medical insurance, medical treatment and medicine, which would change the flow direction of patients to seek medical treatment

Medical service fee was a brand new concept. The setting of medical service fee could reflect the value of doctors' technical services and could effectively guide the flow direction of patients with chronic diseases. The adjustment of medical service fee was only one of hierarchical diagnosis and treatment measures, and other policies and supporting measures were needed. From the current studies, patient access diversion was achieved mainly by means of significantly different tiered pricing and fees, differentiated service positioning between primary care institutions and large hospitals, and differentiated health insurance reimbursement systems [31, 32]. In China, medical insurance reimbursement is one of the most important tools to guide patients to rational medical care, and the behavior of patients' medical needs is regulated and promoted by differentiated medical insurance reimbursement policy. One study showed that the higher the reimbursement rate of a medical institution, the greater its ability to attract residents to medical care [33]. Medical service fees should be designed in combination with measures such as family doctor system, chronic disease management, differentiated reimbursement of medical insurance, long prescriptions for essential medicines, and docking with drug catalogs in secondary and tertiary hospitals in order to coordinate the flow direction of patients with chronic diseases from tertiary hospitals to primary hospitals to further promote the effectiveness of hierarchical diagnosis and treatment. At the same time, the government still needs to strengthen the investment in primary health and the construction of primary service capacity. Although the implementation of hierarchical diagnosis and treatment has achieved some results, there are still many obstacles and problems, and there is still a long way to go [34].

### Research strengths and limitations

Designing the model requires both agent-centric thinking and expertise in medical and health policies, as well as technical support in programming and modeling. So cooperating with programmers was an effective way to acquire programming skills in medical policy ABMS projects [13], In the process of our research, "team scientific collaboration" has been an excellent exercise. At the same time, ABMS required researchers to use computers to predict behavioral dynamics before implementing interventions in the some field [35]. It is a scientific method of trial and error and avoiding policy deviations. In this study, the method of simulation was used to simulate the setting of medical service fees in different levels of hospitals, in order to ensure the scientific and validity of the simulation results, the simulation was checked by comparing the simulation data with the real data.

Many studies on patients' medical choices behavior were hampered by the lack of key factors. Because some important attributes for patients were not easily observed by researchers [36]. There had some limitations in this study, too. The outpatient medical service fee currently implemented in Beijing had four levels: general outpatient, deputy chief physician, chief physician and well-known experts. In order to simplify the model, this experiment only studied the adjustment of general outpatient medical service fee. As a result, the setting of model parameters could not fully match the actual situation of Beijing. In addition, in this study, not all factors that affected patients' choice of medical treatment were included in the model. Therefore, the rules of the residents' medical selection process were slightly simple, and could not reflect its realistic characteristics in detail. In addition, the simulation of medical service fees set the adjustment range. Although the adjustment scope of this study was relatively large, other optimal solutions might be missed.

## Conclusions

This study utilizes the relevant statistics in Beijing and takes hypertension as the entry point to construct a simulation research framework. Through Agent simulation experiments, we explore the impact of the adjustment of medical service fee on patients' choice of medical care. According to the results of the simulation, when adjusting the medical service fee of primary hospitals individually or combined, the proportion of patients attending primary hospitals gradually decreases, the proportion of patients attending tertiary hospitals gradually increases, and there is no significant change in the flow of patients attending secondary hospitals. When adjusting the medical service fees of tertiary hospitals individually or combined, the proportion of patients attending primary hospitals gradually increased, the proportion of patients attending tertiary hospitals gradually decreased, and there was no significant change in the flow of patients attending secondary hospitals. When adjusting the medical service fee for secondary hospitals alone, there was no significant change in the flow of patients through all levels hospitals. Hence, in order to direct the flow of patients to primary hospitals, the medical service fees of primary hospitals should be adjusted individually or combined, so as to give play to the economic leverage of policies.

## Data Availability

The data for this study are part of the overall project and so are not publicly available. Access to the datasets of this study can be directed to the corresponding authors. Basic demographic data of Beijing residents were obtained from the Beijing Municipal Bureau of Statistics. Hospital outpatient data were obtained from the Information Center of the Beijing Municipal Health Commission and the Beijing Community Health Service Management Center. Please contact author for data requests.

## Abbreviations

ABMS: Agent-Based Modeling and Simulation
DCE: Discrete choice experiment
